# Drooling as a Red Flag: Insights From a Case Series in Severe Dermatomyositis With Literature Review

**DOI:** 10.1002/ccr3.71649

**Published:** 2025-12-08

**Authors:** Alireza Mirzamohamadi, Shokufe Sadeghi, Yalda Nilipour, Somayeh Motamed, Vahid Ardestani Rostami, Ahmadreza Jamshidi, Masoomeh Akhlaghi, Hoda Kavoosi, Majid Alikhani, Seyedeh Tahereh Faezi

**Affiliations:** ^1^ Rheumatology Research Center Tehran University of Medical Sciences Tehran Iran; ^2^ Pediatric Pathology Research Center, Research Institute for Children's Health Shahid Beheshti University of Medical Sciences Tehran Iran; ^3^ Neuromuscular Research Center Tehran University of Medical Sciences Tehran Iran

**Keywords:** case series, drooling, dysphagia, refractory dermatomyositis, sialorrhea

## Abstract

Dermatomyositis (DM) is a rare idiopathic inflammatory disease characterized by progressive proximal muscle weakness and distinctive dermatologic manifestations. Although dysphagia is a recognized complication of oropharyngeal muscle involvement, drooling (sialorrhea) is infrequently described and may be a marker of severe disease. This case series reports three adult patients with severe DM who presented with drooling, highlighting diagnostic challenges and therapeutic dilemmas in the management of refractory disease. All patients in the present study exhibited severe DM with prominent dysphagia, leading to drooling. Despite intensive immunosuppressive therapy, including high‐dose corticosteroids, immunosuppressants, intravenous immunoglobulin, and Rituximab, the outcome was disappointing, highlighting the refractory nature of the disease. The patients experienced readmissions following the worsening of dysphagia and drooling, which eventually led to complications like aspiration pneumonia and sepsis and ultimately fatal outcomes. Anti‐Ro52 and Anti‐Nuclear Matrix Protein 2 (NXP2) antibodies correlated with a more severe disease course and poor prognosis. Although rare, drooling in DM may be a clinical marker of severe DM with extensive oropharyngeal involvement and an increased risk for life‐threatening complications. Early evaluation and initiation of intensive combination therapy could improve patient outcomes. Further research is needed to refine treatment protocols for this challenging manifestation of DM patients.

## Key Clinical Message

In adults with dermatomyositis, drooling indicates severe disease and high mortality risk. We recommend comprehensive myositis antibody panels to guide prognosis and treatment intensity. Aggressive combination therapy with a step‐down approach, plus infection prevention, is critical for these patients.

**Abbreviations**
AZA: azathioprine
CA125: cancer antigen 125
CA19‐9: cancer antigen 19‐9
CEA: carcinoembryonic antigen
COVID‐19: coronavirus disease 2019
CPK: creatine phosphokinase
CRP: C‐reactive protein
CT: computed tomography
ECG: electrocardiogram
EF: ejection fraction
EMG: electromyography
ESR: erythrocyte sedimentation rate
FANA: fluorescent antinuclear antibody
Hb: hemoglobin
HCV: hepatitis C virus
HIV: human immunodeficiency virus
HTLV: human T‐cell lymphotropic virus
IVIG: intravenous immunoglobulin
MCV: mean corpuscular volume
MRI: magnetic resonance imaging
NCV: nerve conduction velocity
NGT: nasogastric tube
NXP2: Anti‐NXP2 antibodies
PACs: premature atrial contractions
PAP: pulmonary artery pressure
PCR: polymerase chain reaction
PDN: prednisolone
Plt: platelet count
PVCs: premature ventricular contractions
RO52: anti‐Ro52 antibodies
UGIE: upper gastrointestinal endoscopy
WBC: white blood cell

## 1 Introduction

Dermatomyositis (DM) is a rare idiopathic inflammatory myopathy characterized by progressive symmetrical proximal muscle weakness and distinctive skin manifestations, including facial erythema, V‐sign, heliotrope rash, and Gottron's papules, besides the elevation of the level of muscle‐associated enzymes in serum. Although the pathogenesis of DM remains not fully understood, it is believed to involve autoimmune mechanisms that target striated muscle fibers and skin, leading to inflammation and potentially resulting in myofiber necrosis [1].

In 32.2% of DM patients, involving the striated muscle in the oropharyngeal, laryngeal, and upper esophagus (by reducing pharyngeal contraction and impairment of the upper esophageal sphincter) leads to dysphagia, which can increase the risk of aspiration pneumonia [1, 2, 3]. While the common belief is that upper esophageal sphincter impairment causes dysphagia, studies using VFSS (Videofluoroscopic Swallowing Study) and manometry indicated that impaired pharyngeal muscle contraction due to weakness in the suprahyoid muscles may be more significant [4]. Dysphagia can limit medication intake, worsen prognosis, cause dehydration or prolonged hospital stays, and increase mortality [1]. The more severe form of dysphagia, known as aphagia, may lead to drooling (sialorrhea), a symptom rarely reported in the literature (only six documented cases, all responsive to treatment) [3, 4, 5, 6, 7, 8].

This case series explores the clinical presentations, diagnostic, and treatment challenges in a case series of refractory DM with drooling (as an atypical manifestation). By reviewing these cases, we aim to highlight the complexities of timely diagnosis and management to provide insights into optimizing treatment strategies. Written consent for publication was obtained from living patients or surrogate decision‐makers for deceased patients. Patient data were handled with strict confidentiality.

## 2 Case Presentation

### 2.1 Case 1

#### 2.1.1 Case History and Examination

A 47‐year‐old woman was admitted to our tertiary referral hospital on 1 November 2023 due to severe worsening muscle weakness and odynophagia.

Her symptoms had begun 5 months earlier with flank pain and genital edema. A magnetic resonance imaging (MRI) at that time had shown severe subcutaneous edema in the abdominal walls and thickening of the external oblique muscle. Subsequently, she had developed bilateral wrist and elbow arthralgia with arthritis and painful erythematous rashes on inner thighs, which later appeared on the face, anterior neck, and external surfaces of the arms. She had been hospitalized elsewhere due to progressive proximal muscle weakness in all limbs, to the extent that she was unable to climb stairs and pain. At that medical center, despite elevated CPK (5323 U/L), Electromyography and Nerve Conduction Velocity (EMG‐NCV) studies and muscle biopsy results were normal. Considering normal levels of complements, negative serology tests for lupus (Double‐Stranded DNA, Fluorescent Antinuclear Antibody) and vasculitis (Antineutrophil Cytoplasmic Antibodies), physicians at that hospital had diagnosed her with dermatomyositis and morphea, and she had received methylprednisolone 1 g/day for 3 days. She had been discharged on prednisolone (PDN) 60 mg/day and methotrexate 15 mg/week. However, despite high‐dose corticosteroid therapy, no improvement had been achieved; in fact, her symptoms had worsened and odynophagia had developed, ultimately leading to her referral to our center.

On admission to our center, vital signs were normal. She had a proximal muscle weakness pattern (strength = 2/5) in upper and lower limbs and cervical flexor weakness (strength = 3/5). She exhibited heliotrope rash, facial erythema involving the nasolabial folds, calcinosis on the lateral surfaces of the arms, 2–4 cm purplish erythematous indurated lesions on the lateral and medial sides of the arms, and polyarthritis (right wrist, ankles, and elbows). Neurological, cardiovascular, respiratory, breast, and abdominal physical examinations were normal, and no lymphadenopathy or organomegaly was noted.

#### 2.1.2 Differential Diagnosis, Investigations, and Treatment

Since the patient's condition had worsened over the past 2 weeks despite high‐dose corticosteroid therapy, further evaluation was pursued. Although prior muscle biopsy and EMG‐NCV had been reported as normal, and the cutaneous involvement was atypical, we suspected DM based on clinical and laboratory findings. The lack of response to methylprednisolone pulse suggested possibilities including: (1) Insufficiency of monotherapy (necessitating combination treatment). (2) Paraneoplastic DM, given its known malignancy association. (3) Steroid‐induced myopathy (unlikely, as other DM symptoms would still improve). (4) Infectious/non‐immune myopathies (less probable given characteristic cutaneous/articular involvement).

**FIGURE 1:**
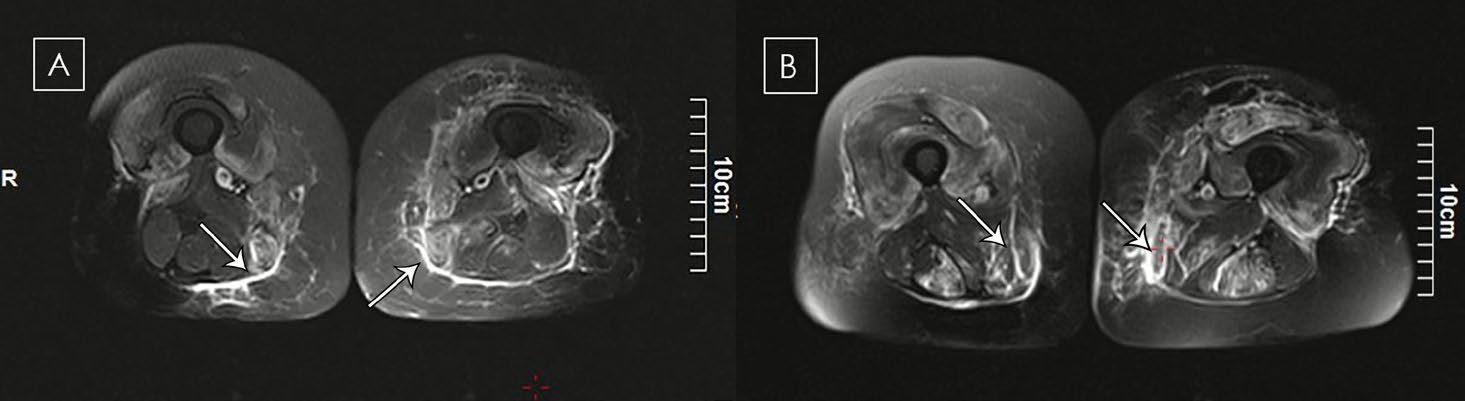
(A) MRI of case 1 revealed mild fatty changes in the sartorius, hamstring, and gracilis muscles. Subcutaneous and soft tissue edema, as well as fascia thickening, are seen in both thighs. (B) There is diffuse, predominant, symmetric, and notable patchy intra‐ and perimuscular edema in both thigh muscles and the lower pelvis. Perimuscular fluid is more prominent along the gracilis and vastus lateralis, as well as along the superficial fascia and minimally along the deep fascia.

**FIGURE 2:**
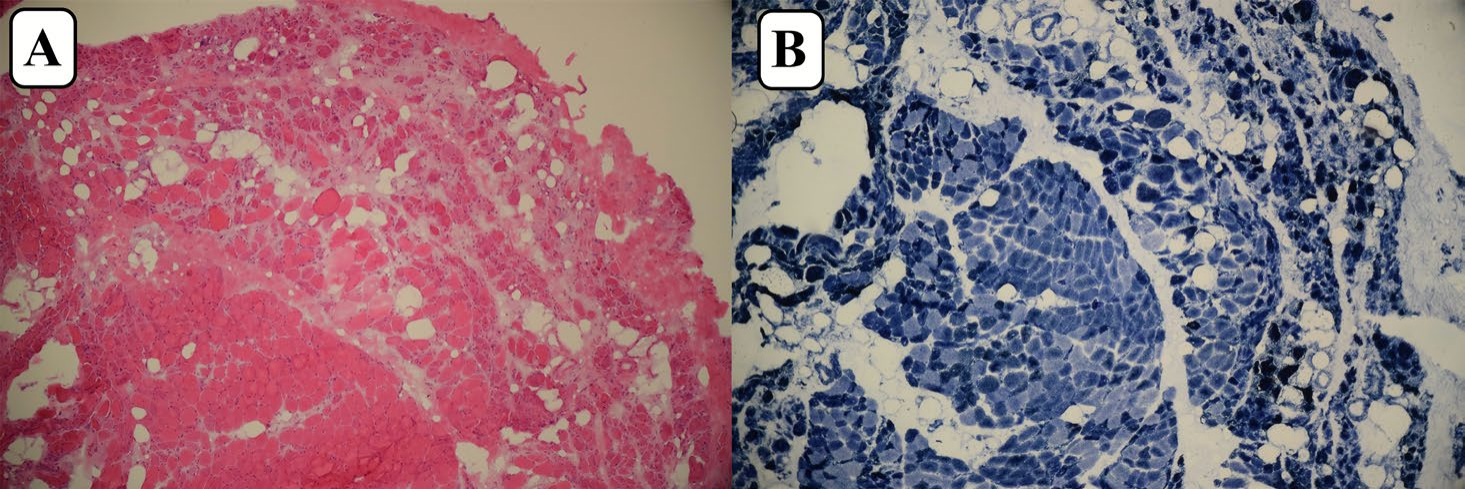
(A) Muscle biopsy of case 1 Revealed patchy severe atrophy, fibrosis, and adipose tissue replacement, indicative of end‐stage muscle pathology without evidence of inflammation (H&E ×100). (B) NADH staining highlighted perifascicular atrophy (×100).

Initial laboratory tests showed leukocytosis, myositis, and elevated liver enzymes (Table 1, first admission). Malignancy workup, including tumor biomarkers (Carcinoembryonic Antigen (CEA), Cancer Antigen 19‐9 (CA19‐9), and Cancer Antigen 125 (CA125)), spiral chest, abdominal, and pelvic Computed Tomography (CT) scans, mammography, gynecologic examination, and occult blood test, was negative. The test results for Influenza A virus subtype H1N1, Coronavirus disease 2019 (COVID‐19) polymerase chain reaction (PCR), Hepatitis B surface antigen (HBsAg), Hepatitis C virus antibody (HCV Ab), and Human Immunodeficiency Virus antibody (HIV Ab) were all negative. Anti‐Ro52 antibody was positive. MRI of the thighs (Figure 1) supported the diagnosis of inflammatory myopathy and contradicted the previously normal biopsy findings from the referring hospital. An experienced pathologist performed a repeat muscle biopsy at our institution, revealing late‐stage severe DM with irreversible sequelae (Figure 2), suggesting poor prognosis for functional recovery.

**TABLE 1:** Summary of patients' selected laboratory test results.

Lab tests	Case 1		Case 2		Case 3		Reference range
	1st A.	2st A.	1st A.	2st A.	1st A.	2st A.	
Wbc (k/micL)	12.19↑/12.7↑	8.55↑/3.5	8.8↑/10.95↑	7.08↑/3.85	12.3↑/19.4↑	23↑/14.4↑	4.5–1.25
Hb (g/dL)	12.1/11.9↓	11.6↓/5↓	7.9↓/10.4↓	10↓/9.4↓	11.9↓/12.3	10.2↓/8.9↓	12–15
Plt (k/micL)	299/176	246/96↓	326/345	150/167	225/224	268/75↓	150–450
ESR (mm/h)	48↑	32↑	37↑	29↑/45↑	10/10	7/36↑	0–15
CRP (mg/L)	11↑	2.7/90↑	10.2↑	9.3↑/4.8	27↑/4.1	26↑/109↑	Up to 6
BUN (mg/dL)	21/18.5	13	18/27↑	24↑/13.4	15/17	16/15	7–20
Cr (mg/dL)	0.6/0.5	0.4↓	0.8/0.9	0.9/0.5	0.9/0.8	0.8/0.9	0.9–1.3
AST (U/L)	261↑/146↑	52↑	260↑/139↑	83↑/44↑	1240↑/558↑	285↑/8800↑	Up to 37
ALT (U/L)	76↑/59↑	27	124↑/114↑	63↑/47↑	418↑/282↑	105↑/6170↑	Up to 40
ALP (U/L)	107/87	122	128/131	139/116	127/138	131/253	80–300
CPK (U/L)	4880↑/1600↑	371↑	4593↑/1069↑	595↑/177	24,100↑/7160↑	4400↑/6200↑	Up to 195
LDH (U/L)	1391↑/890↑	945↑	1396↑/886↑	1345↑/792↑	3570↑/2061↑	1916↑/15,600↑	Up to 480
Aldolase (U/L)	4	4.1	19.7↑	—	3.9	4	0–5
Trop (ng/mL)	Negative	Negative	Negative	Negative	Negative	—	Up to 34
Ca (mg/dL)	8.3↓	8.5	8.7	8.1	8.8	8.9	8.5–10.5
Alb (g/dL)	3.5	3.6	4.1	4.2	3.9	4	3.5–5
P (mg/dL)	3.3	3.1	2.9	4.3↑	3.1	2.8	2–4
Mg (mg/dL)	1.7↓	1.8	—	2.1	1.9/2.1	2/2.1	1.8–2.6
FANA	1/80		1/320		1/160		1/80
Myositis Ab panel	RO‐52 (+++)		RO‐52 (+) and NXP2 (+++)		Negative		

*Note:* ↑ indicates values above the reference range; ↓ indicates values below the reference range/separates repeated test results during hospitalization.

Abbreviations: 1st A., first admission; 2st A., second admission; Alb, albumin; ALP, alkaline phosphatase; ALT, alanine aminotransferase; AST, aspartate aminotransferase; BUN, blood urea nitrogen; Ca, calcium; CPK, creatine phosphokinase; Cr, creatinine; CRP, C‐reactive protein; ESR, erythrocyte sedimentation rate; FANA, fluorescent antinuclear antibody; Hb, hemoglobin; LDH, lactate dehydrogenase; Mg, magnesium; Myositis Ab Panel, myositis antibody panel; P, phosphorus; Plt, platelet; Trop, troponin; WBC, white blood cell.

Treatment with Dexamethasone 12 mg/day, Intravenous Immunoglobulin (IVIG) 120 g, and Rituximab 1 g was initiated to alleviate arthritis and skin lesions. Given the previously normal EMG‐NCV report, a repeat study was done in our hospital, demonstrating irritable myopathic changes. Cardiac and pulmonary workups were normal.

Eight days after admission, the patient was discharged on PDN 80 mg/day, Azathioprine (AZA) 150 mg/day, Hydroxychloroquine 400 mg/day, and a scheduled Rituximab 1 g for 2 weeks later.

Three weeks later, she was readmitted with worsening proximal muscle weakness (1–2/5 in upper and lower limbs and cervical flexors) and drooling. Physical examinations showed tachycardia (PR = 122 bpm), bilateral periorbital edema, and muscle strength decline. Relevant laboratory results are detailed in Table 1 (second admission).

Cardiac workup for suspected cardiomyopathy revealed sinus tachycardia, EF = 50% with septal hypokinesia on echocardiography, and rare premature atrial contractions (PACs) and premature ventricular contractions (PVCs) on Holter monitoring (consistent with DM‐related cardiac involvement).

Despite improvement in muscle enzymes and treatment with high‐dose steroids, IVIG, and rituximab, the muscle weakness progressed, and drooling persisted. Therefore, a neck and brain MRI was done, which excluded structural and neurological etiologies. Finally, dermatomyositis was considered the underlying cause of drooling.

She received dexamethasone (12 mg/day) and IVIG (2 g/kg).

Tachycardia persisted despite anemia treatment, fever management, and beta‐blocker therapy.

Due to odynophagia and immunosuppression, cytomegalovirus esophagitis was suspected and confirmed by PCR. While ganciclovir treatment reduced viral load, drooling persisted.

Later, she developed fever, cough, and hypoxia (O_2_ saturation 88%). Despite repeated Intensive Care Unit (ICU) admission offers, the patient/next of kin declined. Chest CT scan was suggestive of aspiration pneumonia, and bronchoscopy isolated *Pseudomonas aeruginosa* , leading to the initiation of targeted antibiotic therapy. By the third week of hospitalization, new‐onset fever, pancytopenia (White Blood Cell 3500/μL, Hemoglobin 8 g/dL, platelets 96,000/μL), and elevated C‐reactive protein (CRP, 90 mg/L); broad‐spectrum antibiotics were started for suspected sepsis, resulting in fever resolution.

One month into hospitalization, after developing upper gastrointestinal bleeding and a hemoglobin drop to 5 g/dL, she required endoscopic clipping.

Despite the anti‐Pseudomonas antibiotic treatments, the patient's blood culture returned positive for *Pseudomonas aeruginosa* .

#### 2.1.3 Outcome

Unfortunately, the patient did not respond to the treatments provided and, 5 weeks after admission, expired due to respiratory failure on 8 January 2024.

### 2.2 Case 2

#### 2.2.1 Case History and Examination

A 42‐year‐old woman, with no significant past medical history, presented to our tertiary referral center on 31 July 2023, with 3‐month progressive proximal muscle weakness (lower > upper extremities). In physical examination, she had two 3‐cm ulcerated erythematous plaques on the upper sternum and right flank. Proximal weakness (upper limbs 4/5, lower limbs 2/5, cervical flexors 3/5) was evident, while distal strength remained intact.

#### 2.2.2 Differential Diagnosis, Investigations, and Treatment

Based on the patient's history, the primary diagnosis was DM (atypical ulcerative lesions suggested necrotizing variant or overlap syndrome). Alternative considerations included systemic lupus erythematosus with vasculitis, paraneoplastic myopathy (given DM's association with malignancy), infectious myositis (e.g., HIV‐associated), and, less likely, sarcoidosis.

Laboratory tests (Table 1, first admission) revealed markedly elevated CPK (4593 U/L) and negative results for Influenza A virus subtype H1N1, COVID‐19 PCR, HBsAg, HCV Ab, and HIV Ab. EMG‐NCV studies indicated irritable myopathy. MRI of the muscles showed evidence of active inflammatory myositis without muscle atrophy. The muscle biopsy was non‐diagnostic. Further investigations revealed no lung or heart involvement or malignancy with the diagnosis of DM, the treatment was initiated with PDN 1 mg/day/kg. Nine days after admission, the patient was discharged on PDN 60 mg and AZA 50 mg/day.

**FIGURE 3:**
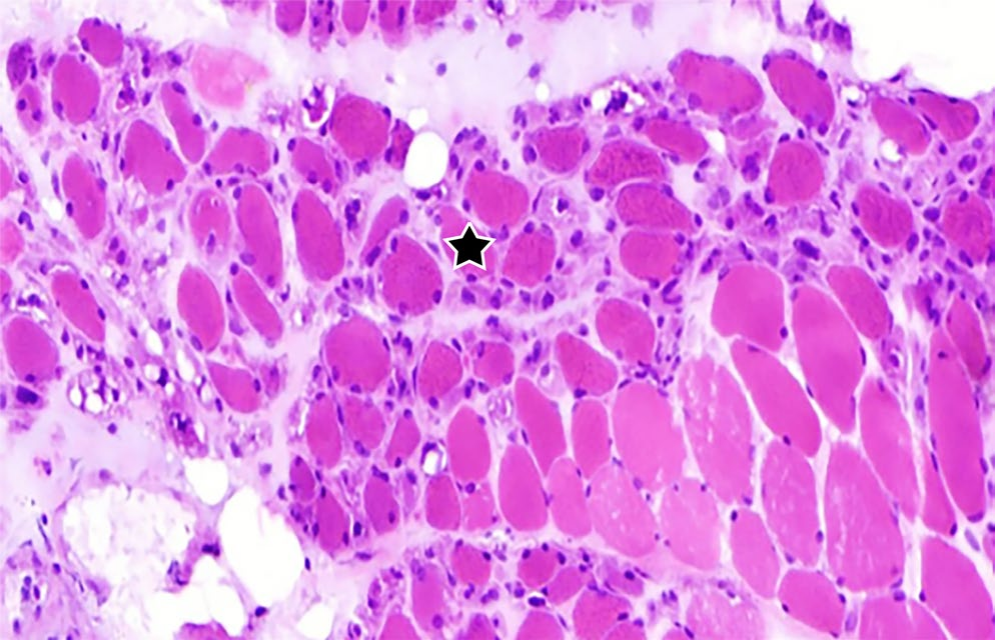
Atrophy of muscle fibers with basophilic degenerative and regenerative fibers, more prominent in the peri‐fascicular region (black star), accompanied by edema of the perimysium, but without inflammation (H&E ×200).

Despite repeated follow‐up recommendations, the patient was lost to follow‐up. One month later, the patient was readmitted due to worsening proximal muscle weakness (proximal upper limbs 3/5 and lower limbs 2/5, and cervical flexors 3/5), dysphagia, and nasal regurgitation of fluids. CPK had decreased to 523 U/L (laboratory findings are in Table 1—second admission). Despite recommendations, the patient and family declined ICU transfer. The patient tested positive for RO52 and anti‐nuclear matrix protein 2 (NXP2) antibodies. A repeat muscle biopsy confirmed DM (Figure 3). The treatment, including IVIG 2 g/kg and dexamethasone 12 mg/day, was initiated.

#### 2.2.3 Outcome

Muscle enzyme levels declined, but weakness still persisted. Progressive drooling developed during hospitalization, followed by aspiration pneumonia, which led to cardiopulmonary arrest 1 month after the second admission.

### 2.3 Case 3

#### 2.3.1 Case History and Examination

A 38‐year‐old man presented in March 2024 with progressive muscle weakness and myalgia in all limbs, starting 2 months earlier, and gradually worsening after a household viral exposure (chickenpox). On admission, he was experiencing new‐onset dyspnea, and there was no skin involvement. Proximal weakness (upper and lower limbs 2/5) was evident, while distal and cervical flexor strength was normal.

#### 2.3.2 Differential Diagnosis, Investigations, and Treatment

Based on the clinical picture, the primary diagnosis included inflammatory myopathies, with polymyositis as the leading consideration. Moreover, the history of viral exposure suggested post‐infectious myositis. The development of respiratory symptoms necessitated evaluation for associated lung disease. The absence of fatigable weakness or ocular symptoms made neuromuscular junction disorders highly unlikely.

Laboratory tests revealed markedly elevated muscle enzymes and liver enzymes, with positive Fluorescent Antinuclear Antibody (FANA, 1:160) (see Table 1—first admission). Myositis‐specific antibodies were negative. The initial treatment included PDN 1 mg/kg. Chest CT demonstrated bilateral mild pleural effusions, passive atelectasis, and chest wall edema. Therefore, antibiotic therapy was started. Cardiac evaluations were normal. Thigh MRI revealed symmetrical abnormal hyperintensity in both thighs and pelvic girdle muscles, with edematous changes in the peri and inter‐muscular fascia.

After 8 days, the patient was discharged on PDN 1 mg/kg and AZA 50 mg/day with arranged biopsy and lab monitoring for dose adjustment, but was lost to follow‐up.

**FIGURE 4:**
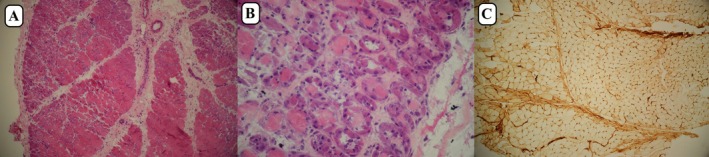
(A) Perifascicular atrophy and degeneration of muscle fibers (H&E ×100). (B) Basophilic degeneration and vacuolation of perifascicular muscle fibers (H&E ×400). (C) MHC1 expression of perifascicular muscle fibers (×100).

One week later, he was readmitted with dysphagia and drooling, along with worsening respiratory distress (O_2_ saturation 75% on room air, hypercapnia), proximal muscle strength rated at 1/5, and 2/5 cervical flexor strength. Laboratory data are outlined in Table 1. Readmission evaluations confirmed severe dermatomyositis via muscle biopsy (Figure 4), and he was transferred to the ICU. Given the fever and the chest CT scan report of aspiration pneumonia with patchy ground glass, he was treated with antibiotics alongside IVIG and Dexamethasone (12 mg/day). Due to respiratory distress and hypercapnia, he was intubated. Bronchoscopy and bronchoalveolar lavage revealed many white blood cells with mixed growth.

#### 2.3.3 Outcome

Despite receiving IVIG and broad‐spectrum antibiotics, the patient expired due to sepsis following aspiration pneumonia.

## 3 Discussion

### 3.1 Overview of the Cases

This case series of three adults with severe DM highlights the importance of timely diagnosis and intervention, especially when symptoms signal an advanced disease. All presented patients exhibited atypical and severe symptoms, including extreme muscle weakness (without typical skin symptoms), dysphagia, and particularly drooling.

Diagnosis was challenging due to an initially normal biopsy and EMG‐NCV, which required repeat testing to correlate with the clinical picture. Notably, Case 1 exhibited inner thigh erythematous rashes, and Case 2 presented with painful and ulcerative lesions, which are not characteristic features of DM (like heliotrope rash or Gottron's papules) and complicate the diagnosis.

All patients developed aspiration pneumonia following drooling and dysphagia and subsequently died. The delayed initiation of combination therapy (resulting from both late referrals to a center with specialists in autoimmune diseases and contraindications to active infection) likely contributed to poor outcomes. Moreover, Case 2's mild initial presentation led us to prescribe oral PDN and AZA, instead of rituximab, but non‐adherence to outpatient follow‐up resulted in a severe relapse, requiring a second hospitalization. In Case 3, rituximab was withheld due to pulmonary infections, and post‐discharge non‐compliance led to readmission and fatal sepsis.

These cases and the documented poor‐prognosis biopsies suggest that drooling can signal advanced dermatomyositis, as it indicates extensive muscle involvement, though a definitive direct link requires further study. Moreover, outcomes emphasize the need for greater awareness and rapid intervention in severe DM with drooling to improve patients' survival.

### 3.2 Drooling: Causes, Evaluations, and Treatment

Drooling, the overflowing of saliva from the mouth, may also result from oral motor dysfunction, inadequate swallowing, a deficit of the oral sphincter, or sialorrhea (e.g., medication side effects). Typically, drooling occurs due to neurological issues, either central (such as cerebral palsy) or peripheral (like facial palsy) [9]. While less common, true hypersalivation can be caused by factors such as oral inflammation, gastroesophageal reflux, or as a side effect of certain medications like clozapine [10]. The oral preparatory and transportation phases of swallowing are often affected, with muscle incoordination hindering the initiation of the swallowing reflex and causing difficulty in smoothly transferring saliva from the mouth to the oropharynx. Factors influencing drooling severity include poor lip seal, muscle hypotonia, macroglossia, dental malocclusion, abnormal posture, emotional state, concentration impairment, reduced oral sensory awareness, and nasal airway obstruction [9]. Therefore, for cases of drooling in DM, consulting a neurologist and an otolaryngologist is essential to exclude other causes.

Identifying the cause of drooling requires a full evaluation, including [9]:

1. Patient history: focusing on the duration and severity of drooling, cognitive status, and any associated neurological conditions.
2. A thorough physical examination of the head, neck, and oral cavity: neurological assessment (particularly cranial nerve function), evaluation of orofacial structures (including labial sphincter competence, dentition, and occlusion), assessment of swallowing mechanisms and salivary gland size/function.
3. Paraclinical studies: barium swallow or fiberoptic endoscopy, lateral neck X‐ray, or CT scan of the head and neck.

Management requires a multidisciplinary approach, including the treatment of underlying causes (like DM). Besides that, other options to reduce drooling include conservative therapies (like oral motor therapy), reducing salivary flow by anticholinergic medications (such as glycopyrrolate and scopolamine), botulinum toxin injections into the parotid and/or submandibular glands, or a combination of these options if needed [9, 10].

Reports on drooling in DM are limited (Table 2), with only six cases documented to date, of which just one involved an adult patient. In contrast, the cases in our series were all adults. The documented cases finally responded to a combination of high‐dose prednisone, AZA, IVIG, cyclophosphamide, methotrexate, and/or hydroxychloroquine. Non‐pharmacologic interventions, including speech therapy and oropharyngeal exercises, were also effective in improving dysphagia and saliva control [3, 4, 5, 6, 7, 8]. However, in our cases, combination therapy with steroids, AZA, IVIG, hydroxychloroquine, and rituximab was not effective.

**TABLE 2:** Summary of case reports on drooling in dermatomyositis.

Study	Patient	Symptoms and involvement	Diagnostic findings	Treatment: Outcome	Final outcome
Zedan et al. (2008) [6]	3.5‐year‐old boy	Peri‐orbital/generalized edema, drooling, Gottron's papules, heliotrope rash, dysphagia, fever, proximal/axial and mild distal muscle weakness, elevated muscle enzymes	EMG: myopathic changes MRI: diffuse edema in pelvis and lower extremities	Methylprednisolone, IVIG, oral prednisolone: only skin rashes improved Discharged with prednisolone, Azathioprine, and NG feeding	After 8 weeks from diagnosis: Partial motor recovery, gastrointestinal improvement, significant decrease in muscle enzymes After 10 weeks from diagnosis: oral feeding, steroid discontinued
Almayouf et al. (2001) [5]	4‐year‐old girl	Drooling, intermittent fever, skin rash, severe muscle weakness (bedridden), respiratory distress Athelectasis, Acidosis	Chest X‐ray: left lung pneumonic infiltration Lung CT scan: pneumomediastinum, bilateral basal atelectasis, and emphysematous bullae in the left lung, which progressed to subcutaneous emphysema	ICU for respiratory issues, severe complications requiring chest tube Prednisone, Methylprednisolone: Fever, Tachycardia IVIG, MTX: gastrointestinal bleeding Cyclophosphamide: Leukopenia Plasmapheresis: temporary muscle improvement, persistent respiratory issues Prednisone, Cyclosporin: recovery in muscle and lung function	Full mobility regained, mild side effects after 8 months and 2 weeks
Lemos et al. (2008) [3]	13‐year‐old girl	Sialorrhea, dysphagia, weight loss, gagging, productive cough, dysphonia, NG tube dependence for feeding (following saliva aspiration)	VFSS: severe oropharyngeal dysphagia, poor swallowing reflex, aspiration	Pulse therapy: general improvement Amitriptyline: to induce xerostomia Speech therapy: Not specified	After 8 weeks: swallowing improved‐moderate dysphagia After 6 months: control saliva aspiration, but was not able to feed exclusively by mouth
Kwon et al. (2018) [4]	53‐year‐old man	Initially: myalgia, generalized weakness, skin rash One month later: Sudden aphagia (even to saliva), generalized edema, drooling	Positive ANA, Negative Anti‐Jo‐1 antibodies VFSS: poor hyolaryngeal movement, delayed reflex, incomplete UES opening EMG: Typical myopathic findings Muscle biopsy: focal perifascicular atrophy and perivascular lymphocytic infiltration in the perimysium and endomysium	Methylprednisolone, Azathioprine: dysphagia worsened after 2 weeks IVIG for 5 days: dysphagia persisted, Pneumocystis jiroveci pneumonia Methylprednisolone, Prednisolone: regular diet after the fourth month Rehabilitative therapy (oropharyngeal exercises, neuromuscular electrical stimulation, and compensatory swallowing techniques): Not specified	Discharged on prednisolone after 2 months Maintained normal swallowing on tapered prednisolone during 3 years of follow‐up
Ruparelia et al. (2018) [7]	8‐year‐old boy	Mild fever, typical skin rash, severe proximal muscle weakness, drooling, poor oral hygiene, food retention in the vestibule Masseter tenderness, limited mandibular movements, with pre‐auricular pain during mouth opening	Posetive Anti‐Jo‐1 autoantibodies EMG: motor axonal neuropathy MRI: inflammatory myopathy	High‐dose prednisone, MTX	Dermatologic/muscular symptom improvement
Shamim et al. (2021) [8]	8‐year‐old boy	^1^ Diffus erythematous rash, body aches, fatigue, bedridden for 2 months Severe proximal muscle weakness, partial neck holding, generalized edema, drooling, dysphonia, and impaired swallowing	Positive ANA EMG: irritable myopathy MRI: post‐contrast enhancement muscle biopsy: perifasicular atrophy and necrosis	Nasogastric feeding Three pulses of Methylprednisolone, subcutaneous MTX, and HCQ for 6 months: stabilized after 6 months and discharged	Admitted emergency department 1 week later
		^2^ Visited emergency department 1 week after discharge with: severe respiratory distress, PRES (seizures, hypertension, vision loss)	Chest X‐ray: Right lung collapse	Mechanical ventilation and suctioning Gastrostomy Tracheostomy IVIG, Methylprednisolone, Cyclophosphamide: gradual improvement in vision and muscle strength 14 days later Parotid botox injection: reduce sialorhea	Remission was noted at the sixth month

Abbreviations: ANA, antinuclear antibody; EMG, electromyography; HCQ, hydroxychloroquine; ICU, intensive care unit; IVIG, intravenous immunoglobulin; MRI, magnetic resonance imaging; MTX, methotrexate; NG, naso‐gastric tube; PRES, posterior reversible encephalopathy syndrome; VFSS, Videofluoroscopic Swallowing Study.

^1^ Patient's fisrt hospitalization.

^2^ Patient's second hospitalization.

### 3.3 Autoantibody Panels and Clinical Course

Myositis‐specific autoantibodies help predict DM severity, malignancy risk, and prognosis. Anti‐Ro52 antibody was positive in Case 1, which is often associated with more severe and rapidly progressive disease and interstitial lung disease [11]. Case 2 exhibited both anti‐Ro52 and anti‐NXP2 antibodies, indicating a complex disease course that may necessitate more aggressive treatment strategies. Patients with positive anti‐NXP2 antibodies often experience more significant muscle weakness rather than skin involvement and are more likely to exhibit symptoms such as myalgia, subcutaneous edema, dysphagia, calcinosis, and even distal muscle weakness [12, 13]. The association between dysphagia and NXP‐2 may explain the observed drooling in Case 2 [11]. In MRI scans, muscle atrophy is more pronounced in patients with positive anti‐NXP2 antibodies compared to those who have negative [13]. This antibody is linked to an increased risk of malignancy [11]. We suggest clinicians prioritize myositis‐specific autoantibody tests as these tests could guide clinicians to make better decisions regarding the intensity of the treatment [11].

### 3.4 Pathology of Muscle Biopsy

Muscle biopsy helps diagnose DM, with the hallmark of perifascicular atrophy [13]. In DM muscle biopsies, myopathic changes, perifascicular atrophy, and regeneration are common, with characteristics of T‐cell‐mediated inflammatory cell infiltration [14]. In our cases, these features were extremely severe (case 1 even showed end‐stage muscle damage with fat replacement, which explains the lack of muscle strength improvement).

When clinicians suspect a disease despite normal results, they should repeat biopsies, as in Cases 1 and 2, where earlier normal biopsies might be due to sampling errors or a patchy distribution of inflammation at the biopsy site. The myositis panel findings can also explain the severity of DM in our cases. In previous studies, Anti‐NXP‐2 was particularly associated with microinfarctions in DM (mostly in acute presentations in children) and regional ischemic immune myopathies [15]. Because of the endomysial capillary loss or membrane attack complex deposition, anti‐NXP‐2 is categorized as a myovascular myopathy antibody [12]. In a study at Johns Hopkins, NXP2‐positive patients had significantly more prominent peri‐fascicular atrophy and peri‐vascular inflammation with less primary inflammation than those who were negative, as in Case 2 (Figure 3). On the other hand, Anti‐Ro52‐positive patients had more primary inflammation than NXP2‐positive patients [16].

## 4 Conclusion

In conclusion, this case series underscores the challenges of managing DM in patients presenting with drooling and dysphagia. We suggest clinicians prioritize diagnostic steps, including accurate biopsy and early comprehensive myositis‐specific antibody panel tests, to guide the treatment approach. Timely initiation of intensive combination therapy, with a step‐down approach as the condition stabilizes, is recommended to prevent irreversible muscle damage. Ensuring patient compliance is also critical to achieving better outcomes. Close monitoring for complications like cardiac involvement and infections is recommended, as they significantly impact prognosis.

Finally, further research is needed to refine treatment protocols, explore emerging therapies, and optimize strategies for managing refractory DM, particularly in severe cases with atypical symptoms.

## Author Contributions

**Alireza Mirzamohamadi:** visualization, writing – original draft, writing – review and editing. **Shokufe Sadeghi:** investigation, validation, writing – review and editing. **Yalda Nilipour:** visualization, writing – review and editing. **Somayeh Motamed:** investigation, writing – review and editing. **Vahid Ardestani Rostami:** investigation. **Ahmadreza Jamshidi:** project administration. **Masoomeh Akhlaghi:** project administration, supervision. **Hoda Kavoosi:** supervision. **Majid Alikhani:** supervision. **Seyedeh Tahereh Faezi:** project administration, supervision, writing – review and editing.

## Funding

The authors have nothing to report.

## Ethics Statement

The authors have nothing to report.

## Consent

Written informed consent was obtained from patients or, in cases where the patient is deceased, from their legal guardians or next of kin, for the publication of this case series.

## Conflicts of Interest

The authors declare no conflicts of interest.

## Data Availability

Data available on request from the authors.
